# Pennsylvania Hospital—Service of Dr. G. W. Norris*This and some other cases yet to be reported were treated under Dr. Peace, whose term of duty expired in December.

**Published:** 1842-02-26

**Authors:** 


### CLINICAL REPORTS.

### *Pennsylvania Hospital—Service of Dr. G. W. Norris.**

CASES REPORTED BY DR. E. HARTSHORNE, RESIDENT.

1. *Double compound fracture of the tibia and fracture of the fibula.*—James W., aged 38, entered Nov. 6th, with a compound

*This and some other cases yet to be reported were treated under Dr. Peace, whose term of duty expired in December.

fracture of both bones of the left leg and severe contusion of the right, caused by the fall of a heavy bank of earth upon the parts. Two oblique fractures four inches apart, the upper one about three inches below the tubercle, were presented in the tibia, separating a fragment which was forced backwards to the depth of nearly half an inch. A depression corresponding to the amount of posterior displacement was thus formed in the spinous ridge of the broken bone, beginning at the upper and terminating abruptly at the lower points of fracture. When first examined, about three hours after the accident, much tumefaction of the parts adjoining the seat of injury had supervened; two small openings, from which freely issued arterial and venous blood, were found on the inner face of the limb, apparently communicating with the lower fracture. Well marked emphysema for the space of two or three inches around the apertures was presented. Spasm of the muscles repeatedly occurred during the examination and subsequent reduction of the fragments, the adjustment of the pillow covered with bran, and of the simple fracture box with which the limb was dressed. A moderate opiate was given, and the spasm subsided soon after the reduction and dressing of the limb. Although the depressed fragment was, with much difficulty elevated from the position into which it had been forced, the operators were unable to restore it exactly to its proper place. A slight inequality, therefore, unavoidably remained for a short distance in the anterior edge of the bone. In other respects, however, the limb preserved a perfectly straight position. Crusts having formed in a short time over the bleeding orifices, the hæmorrhage ceased. The emphysema disappeared in two or three days, and in about six days the external wounds had entirely cicatrized, converting the injury into a simple fracture. The inflammation of the limb, was treated with the lead water lotion and disappeared in ten days. The heel, which in this case was extremely sensitive, while there was a great tendency in it to sink and displace the lower fragment, was well supported with bran, which by its uniformity of pressure presented an excellent resisting surface. At the expiration of two weeks the bran was omitted, cotton wadding being substituted under the heel or under the tendon just above it, while the limb was elevated upon an inclined plane in order to lessen the disposition to sinking of the foot, and to consequent anterior angular displacement. The man, restless by temperament, and impatient under his affliction, was rendered still more so, by repeated attacks of rheumatism, from which he suffered more or less during nearly the whole period of his confinement, and also from diarrhœa of which he had at least two severe paroxysms while under treatment. Firm union was established, however, in fifty days. The fracture box was laid aside, the limb being protected by a bandage, at the end of the eighth week. Passive motion of the knee and ankle was then daily practised, and in the course of ten days longer the patient was allowed to walk about on crutches. Discharged Jan. 24, cured, with some rigidity of the ankle joint still remaining.

*Remarks.*—This fracture was a severe one in its character and complications; yet although the subject of it, naturally irritable in temperament, was provokingly disturbed and to a certain extent reduced by more than one attack of diarrhœa, worried with the repeated onsets of an old rheumatic affection, and obliged nearly throughout the treatment to have the limb considerably elevated—that being the only position tolerable to him—still, the consolidation steadily advanced, and was completed within a reasonable period. The case also exemplifies a valuable property of Dr. J. R. Barton's bran dressing, so often resorted to in this house—one to which the attention of the reporter had been previously called by Dr. Reynell Coates. I refer to its utility, not only in soaking up the effused blood, but in forming an artificial scab over the bleeding apertures, and thus closing them until a natural cicatrix has been formed.

[As Dr. Hartshorne has quoted us in the foregoing article, for an observation upon the effect of the bran dressing, as a means of occasionally converting a compound fracture into a simple one, we refer the reader to the leading article in the original department of this number for some further remarks on the subject.]

